# How Reliable Is In Silico ADMET Profiling? A Comparison of Tools Complementing Experimental In Vitro Analyses of Newly Developed Breast Cancer Therapeutics

**DOI:** 10.3390/biology15212024

**Published:** 2026-11-06

**Authors:** Dominika Kołodziej-Sobczak, Szymon Obertas, Łukasz Sobczak, Krzysztof Z. Łączkowski

**Affiliations:** 1 Department of Chemical Technology and Pharmaceuticals, Faculty of Pharmacy, Collegium Medicum, Nicolaus Copernicus University, Jurasza 2, 85-089 Bydgoszcz, Poland; 2 Department of Pharmacodynamics and Molecular Pharmacology, Faculty of Pharmacy, Collegium Medicum, Nicolaus Copernicus University, Jurasza 2, 85-089 Bydgoszcz, Poland; 3 Galenica SA, Untermattweg 8, 3027 Bern, Switzerland; szymonobertas@gmail.com; 4 Hospital Pharmacy, Emil Warmiński Clinical Hospital of PBŚ, Szpitalna 19, 85-826 Bydgoszcz, Poland

**Keywords:** drug development, medicinal chemistry, anticancer drugs, PTP1B inhibitors, ADMET profiling, in silico screening

## Abstract

In silico analyses, including absorption, distribution, metabolism, excretion, and toxicity (ADMET) profiling and drug-likeness evaluations, constitute powerful tools in modern drug discovery. These computational methods enable high-throughput screening of molecular libraries to assess therapeutic potential while identifying unfavourable pharmacokinetic and toxicological profiles early in the development process. To bridge the gap between computational models and empirical data, this study evaluates the reliability and limitations of predictive ADMET profiling by comparing in silico outputs across multiple platforms and referring them to available in vitro experimental data. The empirical dataset comprises recently published in vitro assays focusing on inhibitors of the protein tyrosine phosphatase 1B (PTP1B) enzyme. ADMET predictions were generated using three widely utilized, non-commercial platforms: SwissADME, ADMETlab 3.0, and admetSAR 3.0. The comparative analysis encompassed physicochemical parameters (MW, log*P*, TPSA), medicinal chemistry alerts (PAINS, ALARM NMR), absorption metrics (Caco-2, HIA), and multiple toxicity endpoints (Ames mutagenicity, DILI, hERG inhibition, nuclear receptor signaling, and mitochondrial toxicity). Significant differences observed in toxicity predictions across the platforms highlight the critical necessity of employing multi-tool predictive strategies to obtain a reliable and comprehensive outlook.

## Simple Summary

Computer tools for ADMET (absorption, distribution, metabolism, excretion, and toxicity) profiling are now essential for designing new drugs. They allow scientists to test many chemical compounds quickly, avoiding long, expensive, and potentially dangerous laboratory experiments by identifying unfavourable drug traits early. To bridge the gap between new computer methods and traditional laboratory tests, this study evaluates the reliability of ADMET computer profiling by comparing virtual results generated to complement prior experimental data. The experimental data were sourced from recently published studies on anticancer drugs that block the protein tyrosine phosphatase 1B (PTP1B) enzyme. We performed the computer analyses using three popular, free, and easy-to-use tools: SwissADME, ADMETlab 3.0, and admetSAR 3.0. Finally, this article compares and discusses the main similarities and differences between these methods.

## 1. Introduction

According to the latest sources, introducing a new drug to the market costs approximately $1.1417 billion. This figure accounts for both direct development expenses and the exploratory research required—processes that are frequently burdened by high failure rates [1]. Given this immense financial scale, it is not surprising that increasing emphasis is placed on increasingly accurate screening before the drug reaches the distant and highly expensive Phases II and III of clinical trials [2,3].

A significant problem of drug design is the need to achieve an ideal balance between the pharmacodynamic mechanism of action, which can be planned, and the more difficult-to-predict pharmacokinetic profile, which affects therapeutic safety. It is this challenge that causes most drug withdrawals from the market [4]. Complications resulting from unfavourable pharmacokinetics are complex in nature and pose a significant challenge to the pharmaceutical industry. While pharmacokinetic complications manifest as unanticipated toxicity during Phase I clinical trials, Phase II failures are predominantly driven by a lack of efficacy, accounting for over 50% of candidate discontinuations [5]. Even with extensive in vitro preclinical animal testing frequently fails to predict human outcomes due to interspecies differences in drug biotransformation [6]. Consequently, traditional animal models exhibit a relatively low predictive value for human efficacy and safety [7]. It is therefore justified to apply the “3Rs principle” (replacement, reduction, and refinement) [8,9,10], which allows for reducing the dependence on animal testing in predicting parameters such as absorption, distribution, metabolism, excretion, and toxicity (ADMET) for newly developed drugs. Artificial intelligence (AI)-supported alternatives are increasingly used for this purpose, as they are more and more accurate, thus ensuring high credibility of the research conducted [11].

Recent reports confirm that AI-supported in silico analyses accelerate drug development by successfully predicting the neurotoxicity of drug candidates for Alzheimer’s and Parkinson’s diseases [12]. Current predictive models are so accurate that they demonstrate superior accuracy (89%) in evaluating the cardiotoxicity of 62 antiarrhythmic molecules compared to traditional rabbit models (75%) [13]. Moreover, in a comprehensive systematic review, Mittal et al. compiled information confirming that simulation models provide adequate reliability to reproduce the results of toxicological and pharmacodynamic studies obtained using animal models for cardiovascular, oncology, diabetes, and infectious diseases drugs [14]. Consequently, regulatory bodies such as the U.S. Food and Drug Administration (FDA) have taken decisive steps to permit the replacement of animal testing with AI-assisted simulations in preclinical evaluations [15]. Beyond the clear ethical advantages, this transition significantly enhances the cost-efficiency of drug development [16,17]. This is due to the possibility of early cessation of the development of faulty molecules, before they even reach the stage of molecule synthesis [18,19]. This proactive termination prevents substantial financial losses on candidates whose pharmacokinetic profiles fail to meet drug-likeness criteria or trigger structural toxicity alerts. The entire drug development process can also be faster when in silico pharmacokinetic and toxicological analyses are implemented, because large computational models allow for efficient analysis of entire libraries of molecules, which is unattainable when using animal models, a factor that has been documented to increase the success rate in drug development processes for innovative drug molecules [20].

However, computational models are not without limitations and should be evaluated critically. Chief among these limitations is environmental and physiological variability, which traditional animal models naturally capture more effectively. Furthermore, there is a gap in the quality of evidence for toxicity prediction based on AI and machine learning ADMET profiling tools and platforms regarding rare idiosyncratic toxicity [21,22,23,24]. Consequently, predictions generated by these tools carry a notable risk of false-positive or false-negative outcomes [25]. This vulnerability stems from complex interindividual variability [26], the challenge of predicting the biological activity of downstream metabolites, and the intrinsic requirement of a whole-organism context to manifest specific toxicological endpoints [27]. Furthermore, predictive accuracy depends on the underlying methodology, typically categorised into rule- or statistical-based, machine learning-based, and graph-based approaches. Each relies on training datasets of varying quality and chemical diversity [28]. Each of them is based on training data of varying quality and a specific representativeness; when evaluating novel, unprecedented chemical entities—a primary application of these platforms—the molecules may fall outside the established applicability domain of the model [29]. Finally, the reproducibility and robust validation of results within clinical or in vitro conditions, rather than solely within isolated, static datasets, remain critical limiting factors [14].

Balancing the clear advantages of AI-assisted methods against their inherent limitations, this study evaluates newly synthesised protein tyrosine phosphatase 1B (PTP1B) enzyme inhibitors acting as anticancer drugs, published in recent years, and performs ADMET profiling for them. The importance of the overexpression of this enzyme in the pathogenesis of numerous human diseases, including cancers, has emerged as a focal point in contemporary oncology [30,31]. Particular emphasis was placed on compiling datasets related to breast cancer therapeutics. In a broader sense, the presented work aims to critically assess the reliability, applicability, and limitations of in silico platforms, ultimately addressing a fundamental question: how reliable is in silico ADMET profiling?

## 2. Materials and Methods

### 2.1. ADMET Profiling Tools Used in This Study

There is a multitude of databases and tools for profiling ADMET parameters, which are all useful in drug discovery. Depending on the sources—and, more importantly, users’ specific expectations and requirements—estimates suggest that there are from 34 [32] to over 50 [33,34] prediction tools available. They differ not only in the number of endpoints they cover but also in the algorithms they employ. Regarding the number of endpoints, ADMETlab 3.0 [35] and admetSAR 3.0 [36] are the clear leaders, each covering 119 endpoints. However, they differ significantly in the underlying models driving them; consequently, they exhibit distinct patterns in the results they yield—a factor worth considering when selecting a platform to suit one’s needs. Both rely on deep learning architectures—specifically, graph neural networks (GNNs). ADMETlab 3.0 is based on a directed message passing neural network (DMPNN) combined with molecular descriptors, whereas admetSAR 3.0 utilises CLMGraph [28]. Each model learns and processes information differently; ADMETlab 3.0 allows for more precise modelling of molecular topology and is unsurpassed in prediction stability. Conversely, admetSAR 3.0—originally trained using ‘contrastive learning’ to identify differences and similarities among unlabelled molecular datasets—proves invaluable and more precise in drug design, particularly when dealing with innovative, previously unknown moieties and scaffolds. Both tools are therefore highly useful during the initial screening of ADMET parameters in the development of new molecules [28]. A case study evaluating the agreement between experimental results for erlotinib (a tyrosine kinase inhibitor anticancer drug) and data from 18 different predictive tools concluded that ADMETlab 3.0 offers excellent precision and accuracy; this, combined with its versatility regarding endpoints [32], accounts for its popularity. In another study, the use of admetSAR 3.0 yielded high result concordance for log*P* and demonstrated the highest level of agreement among all the tools for parameters such as plasma protein binding and metabolic profile evaluation. The third tool used for comparison presented in this study was SwissADME [37]. It relies on a rule-based support vector machine (SVM)—a model trained on extensive datasets that uses fixed rules to determine whether a given molecule meets specific criteria. Its speed and visual data presentation—featuring the proprietary BOILED-Egg (Brain or Intestinal Estimated Permeation) graphical model [38] and a bioavailability radar chart—make it highly user-friendly. Furthermore, it ensures a strong correlation between experimental and predicted results [28], making it one of the most frequently used tools in research, particularly during the preliminary screening stage. Additionally, all three predictive tools are free and require no additional software downloads. These factors led to the selection of these three platforms for the analysis presented below.

### 2.2. Analysed Compounds

In this study, thirty-five molecules (listed in Table 1) were analysed. To ensure data transparency, reproducibility, and rigorous curation, a multi-step workflow was employed for the compilation and standardisation of the molecular dataset. Initially, a total of 35 compounds were retrieved from source publications based on two strict elemental inclusion criteria: (i) demonstrated inhibitory activity against protein tyrosine phosphatase 1B (PTP1B), and (ii) experimentally verified cytotoxic activity against the MCF-7 cell line, which represents the most common breast cancer subtype—with positive immunohistochemical expression of progesterone and estrogen receptors, the absence of human epidermal growth factor receptor 2 (HER2) protein overexpression, and the lack of amplification of the *ERBB2* gene encoding it. Articles were excluded when the information presented was insufficient for a quantitative assessment, when appropriate comparisons were lacking, or when the findings were expressed solely in qualitative terms (e.g., binary statements indicating whether a compound was active or inactive), without numerical values that would allow for mathematical analysis.

To maintain uniformity and ensure comparability across different sources in the literature, all biological endpoints were standardised, with half-maximal inhibitory concentration (IC_50_) metrics expressed in a common unit of µM. Prior to in silico profiling, chemical structures underwent a rigorous standardisation protocol. Molecules were sourced in the stereochemical configurations defined in the original reports. Two-dimensional structures were sketched via the Marvin JS molecular sketcher integrated within the SwissADME interface [37] and converted into raw SMILES strings. To ensure a consistent and standardised dataset for computational evaluation, structural normalisation and canonicalization were verified using the PubChem Standardization Service [45]. This protocol automatically added hydrogen atoms, stripped counter-ions (salts), and resolved minor tautomeric or stereochemical configuration nuances. Ultimately, this process generated standardised canonical SMILES, rearranging the textual format to remain invariant to the initial atom ordering, which were then utilised as the uniform input for all compared profiling tools.

### 2.3. Statistical Analysis

Statistical analysis was performed using parameters such as Cohen’s kappa coefficient, Pearson’s correlation coefficient, and Spearman’s rank correlation. Cohen’s kappa coefficient was determined using an online GraphPad calculator [46], while the other coefficients were calculated using an online Pearson+ calculator [47].

## 3. Results and Discussion

### 3.1. Physicochemical Properties of the Analysed Compounds

Physicochemical parameters—such as molecular weight (MW), lipophilicity (log*P*), and topological polar surface area (TPSA)—were analysed using ADMET prediction platforms (see Table 2). These parameters enabled the prediction of drug-likeness and oral bioavailability in accordance with Lipinski’s Rule of Five and Veber’s rules, as they allowed for the assessment of a molecule’s ability to cross biological barriers, undergo intestinal absorption, and penetrate target cells—aspects that are discussed in subsequent sections of the article.

When assessing the agreement between tools, each parameter was considered individually. The correlation between log*P* and TPSA was evaluated using both the Pearson correlation coefficient and Spearman’s rank correlation (see Table 3), revealing very strong positive correlations for all values obtained in this comparison through the use of ADMET profiling tools.

In the case of MW, the values showed a high degree of consistency across the various tools, as this is an absolute parameter. TPSA results also exhibited strong agreement; however, one tool—SwissADME—consistently yielded higher values. Although TPSA does not allow for the objective prediction of a designed molecule’s activity, it does enable an estimation of the molecule’s potential to permeate biological membranes. Consequently, due to the high consistency of the resulting predictions, it serves as a valuable and reliable component of drug design. However, the most significant differences were observed in the case of log*P* values. The highest values were consistently obtained using the admetSAR 3.0 tool. The mean range across all molecules was 1.46 ± 0.75—a substantial figure resulting from the diverse algorithms employed by the various tools. Nevertheless, despite these differences, each tool enabled an assessment of the trend in a given molecule’s log*P* values. This facilitated the evaluation of potential cell membrane permeability, thereby offering valuable guidance regarding the necessity to further optimize molecules with excessively high log*P* values. This situation was particularly well demonstrated with Compounds 20–35 (with log*P* values ranging from 5.47 to 8.56, depending on the tool used). While these molecules demonstrated a high efficacy in inhibiting the isolated enzyme, the results obtained using MCF-7 cell lines—though still favourable—were noticeably worse than those from isolated enzyme assays. This difference likely arose from the favourable fit of these lipophilic molecules into the enzyme’s lipophilic pockets and allosteric sites. However, this same structural characteristic impaired the permeation of biological barriers (such as cell membranes) due to high membrane retention, thereby reducing bioavailability and, consequently, diminishing the ability to inhibit cell line proliferation. Furthermore, such characteristics can compromise safety in obese patients due to drug accumulation in adipose tissue, which may prolong the elimination half-life [48], and may result in non-specific plasma protein binding, ultimately narrowing the drug’s therapeutic index [49].

By comparing experimental data with in silico results, we observed a trend in which the best optimised molecules (MW < 450 Da, log*P* in the 2.0–4.0 range) exhibited the lowest IC_50_ values for inhibiting breast cancer cell line proliferation; this highlighted the benefits of routine use of predictive tools during the drug design process.

Despite disagreements in the absolute values reported by various ADMET profiling tools, there was a high degree of consistency in their predictions; this ensured reliability and supported their use during the early stages of drug design and development to prevent the synthesis of molecules with suboptimal properties resulting from unfavourable physicochemical profiles.

### 3.2. Medicinal Chemistry Alerts

The PAINS (Pan-Assay Interference Compounds) and ALARM NMR (a La assay to detect reactive molecules by nuclear magnetic resonance) modules, designed to identify interfering compounds, were analysed (see Table S1 in the Supplementary Material). The ADMETlab 3.0 and SwissADME platforms both featured the PAINS module, and the results obtained from them showed 100% agreement. This module is useful for the preliminary assessment of whether molecules contain structural fragments previously reported to yield false-positive results. The ALARM NMR module is based on a different principle, relying on the monitoring of ^13^C chemical shift changes in the human La antigen caused by the reactivity of the analysed molecules [50]. Among the tools employed, only ADMETlab 3.0 featured it. However, it offered significant added value by allowing alerts to be cross-referenced with in vitro data. The reliability of ALARM NMR—specifically regarding the prevention of both false-positive and false-negative results—has already been evaluated by Huth et al. [50]. Its application is grounded in the fact that ALARM NMR enables the detection of non-specific binding to the molecular target [51]. This makes it possible to determine whether a promisingly low IC_50_ value observed in isolated enzyme assays is merely an artefact. The results demonstrated that neither proper structural optimisation nor a reduction in the number of ALARM NMR alerts directly translated to a narrowing of the gap between the compound concentration required to inhibit the isolated enzyme and the concentration needed to inhibit proliferation of cancer cell lines. Nevertheless, the ALARM NMR module provided feedback regarding a given molecule’s predisposition for aggregation, non-specific binding, and non-specific disruption of cellular structures. Consequently, molecules that exhibited activity as inhibitors of isolated enzymes and against cell lines—yet triggered multiple ALARM signals—might have operated via a mechanism other than binding to the enzyme’s catalytic site, or their biological activity might have been enhanced by unintended chemical reactivity.

Furthermore, it is crucial to acknowledge that while a correlation exists between isolated PTP1B inhibition and reduced cancer cell proliferation, this does not unequivocally establish direct target-mediated cytotoxicity. As highlighted by medicinal chemistry alerts evaluation and potential ALARM NMR/PAINS liabilities, cellular antiproliferative effects can also be modulated by non-specific mechanisms, off-target interactions, or unintended chemical reactivity. Therefore, although these compounds serve as valuable structural leads, confirming intracellular target engagement through complementary biophysical or cellular assays (such as cellular thermal shift assays, CETSA) remains an essential next step to definitively associate isolated enzyme inhibition with the observed in vitro anticancer efficacy.

### 3.3. Absorption

Parameters such as the human epithelial colorectal adenocarcinoma cell line (Caco-2) permeability test and human intestinal absorption (HIA) were compared across ADMET profiling tools. A table presenting the outcome of ADMET profiling tools comparison is provided in the Supplementary Material (see Table S2).

An analysis of the Caco-2 permeability test results revealed a Pearson’s *r* of 0.623847 (see Table 4), suggesting a strong positive linear relationship between the two platforms (admetSAR 3.0 and ADMETlab 3.0). However, compounds **11**, **14**, and **17** were identified as potential outliers (or high-leverage observations) and were therefore excluded from the calculations.

Caco-2 permeability test enables the estimation of oral drug absorption in intestinal epithelial cells. Values ranged from −5.71 to −4.49 for ADMETlab 3.0 and from −5.17 to −4.07 for admetSAR 3.0. The difference between the tools ranged from 0.02 to 0.76, indicating a high level of agreement between the obtained results. Drawing conclusions from the generated data, we concluded that while the obtained results would be satisfactory for intravenous drug formulations, structural modifications to the tested compounds would be required if they were to be used as oral anticancer drugs. However, establishing a viable delivery route would still require comprehensive experimental profiling encompassing aqueous solubility, chemical and metabolic stability, in vivo pharmacokinetics, and advanced formulation-specific parameters.

A comparison of the HIA values revealed that the choice of profiling tool can lead to results that are sometimes even contradictory. While ADMETlab 3.0 and admetSAR 3.0 use probability values ranging from 0 to 1, SwissADME employs a binary classification system, categorizing absorption as either “High” or “Low”. The level of agreement between ADMETlab 3.0 and admetSAR 3.0 did not allow for definitive, objective conclusions; the differences between these tools ranged from 0.01 to 0.99. The level of agreement between the obtained results was assessed by calculating Cohen’s kappa coefficient. The results indicate a “slight agreement” between SwissADME and ADMETLab 3.0 (see Table 5), but a complete lack of agreement between SwissADME and admetSAR 3.0 (see Table 6), as well as between admetSAR 3.0 and ADMETLab 3.0 (see Table 7).

This highlighted the need for considerable caution and an understanding of the criteria used to set thresholds and binary categories when selecting a tool. Interestingly, compounds exhibiting the lowest IC_50_ values in tests encompassing the MCF-7 cell line (below 10 µM) were very frequently categorised as having poor intestinal absorption. This observation underscores a significant issue regarding a pattern often encountered in the drug design process. Great emphasis is placed on achieving increasingly lower enzyme-inhibitory concentrations, whereas some of this effort would be better spent on optimising the molecule’s absorption characteristics and other pharmacokinetic properties, which would enhance both its safety and efficacy.

### 3.4. Toxicity

Evaluating predicted organ toxicity models is of paramount significance when characterising newly designed drug candidates. The selectivity index (SI)—determined by the ratio of the IC_50_ for healthy cell lines to the IC_50_ for diseased cell lines (see Table S3 in the Supplementary Material)—was categorised for all of the compounds. Values with an SI < 2 are categorised as low selectivity, which may cause toxicity; values between 2–10 are categorised as moderate selectivity; and those above 10 are categorised as high selectivity with a safe profile apart from the target cell line. It should be emphasised that experimental in vitro data obtained from the literature are characterised by methodological heterogeneity, particularly regarding the non-toxic control cell lines (such as HaCaT, Vero, HSF, and HUVEC) used to assess baseline toxicity. Because these control models possess distinct physiologies and metabolic capabilities, a direct quantitative comparison of raw Selectivity Index (SI) values across different series of compounds introduces systematic error. To mitigate this biological variability and prevent the misinterpretation of data, SI values have been qualitatively categorised into specific safety levels, rather than being treated solely as continuous variables. This experimental discrepancy highlights a significant advantage of incorporating multi-tool in silico ADMET profiling. Computational predictions for endpoints—such as drug-induced liver injury (DILI), blockade of the human ether-a-go-go-related gene (hERG) potassium channel (a measure of cardiotoxicity), or mutagenicity in the Ames test—are generated using standardised mathematical models under identical algorithmic conditions for all 35 molecules analysed. Consequently, in silico screening provides a consistent, objective benchmark for comparing pharmacokinetic and toxicological profiles, effectively neutralising differences stemming from inconsistent laboratory protocols.

The application of ADMET profiling tools revealed that the evaluated substances pose a potential risk of causing various serious health consequences (see Table S4 in the Supplementary Material). A trend was observed indicating an increased risk of mitochondrial toxicity and DILI for the molecules showing the highest activity against the MCF-7 cell line (compounds **3**–**7** and **9**, with IC_50_ values below 10 µM). In these instances, there was complete agreement among the ADMET profiling tools. The agreement between admetSAR 3.0 and ADMETLab 3.0 was evaluated for the Ames test using Cohen’s kappa (see Table 8), yielding a *κ* value of 0.374, which indicates “fair agreement”. This suggests that the level of consistency is relatively low. The correlation of toxicity parameters—such as DILI, the Ames test, hERG, and effects on the Nuclear Receptor-Androgen Receptor (NR-AR) and Nuclear Receptor-Estrogen Receptor (NR-ER)—was examined using statistical methods, including Pearson’s *r* and Spearman’s rank correlation (see Table 9). These analyses revealed strong or very strong positive correlations for DILI and hERG (at 10 µM). The correlation for the Ames test was weaker, showing a “moderate positive correlation”, which is consistent with the result obtained from the Cohen’s kappa analysis. Conversely, “weak” to “very weak” correlations were observed between the ADMET profiling tools for NR-AR and NR-ER, indicating that the analysis of these endpoints should be approached with a high degree of caution.

Regarding mitochondrial toxicity, the results obtained from the two tools cannot be directly compared because they utilise different measurement endpoints. ADMETlab 3.0 yields results focused on changes in mitochondrial membrane potential, whereas admetSAR 3.0 indicates a broader range of damage, encompassing overall mitochondrial dysfunction. Consequently, in terms of toxicity, ADMET profiling tools should currently be viewed as providing indications rather than definitive determinations of these parameters. Nevertheless, comparing results from both tools for mitochondrial toxicity analysis reveals consistent patterns; obtaining identical results with similar probability levels from both programs provides significant information about the compound under study. A low probability across both platforms suggests a molecule free from mitochondrial toxicity. Mixed results may indicate mitochondrial damage or destruction resulting from membrane potential disruption or other, unrelated mechanisms. Conversely, high probability scores from both tools serve as a valuable indicator that the molecule may still require optimisation. Agreement between the tools regarding NR-ER activity in the context of breast cancer could also provide valuable information. Most breast cancers exhibit positive estrogen receptor expression (as seen in the analysed MCF-7 cell line). This is a crucial insight, as it allows for the early-stage elimination of drug candidates that might promote adverse estrogenic effects in hormone-dependent cancers. Therefore, ensuring convergence among ADMET profiling tools for endpoints such as NR-AR and NR-ER is a crucial aspect that should be prioritised. Moreover, recent advances in computational medicinal chemistry emphasise the critical role of integrating multi-aspect computational workflows—encompassing Density Functional Theory (DFT), molecular docking, and comprehensive ADMET profiling—to evaluate newly synthesised heterocyclic scaffolds with high accuracy. Aligning with these contemporary methodological standards, our findings reinforce the necessity of endorsing experimental biological data with robust computational predictions to facilitate the early-stage development of targeted therapeutics [52].

### 3.5. Limitations of the Current Study and Future Perspectives

Finally, we must acknowledge a key limitation of this study: direct experimental ADMET data for the evaluated compounds are currently unavailable. Consequently, while our results demonstrate a high consensus among the utilised prediction tools and highlight plausible associations between predicted physicochemical properties and biological activity, this study does not establish the absolute predictive accuracy of these computational models for the examined endpoints. Future in vitro and in vivo studies are required to empirically validate these in silico findings. While physicochemical profiles and computational predictions provide valuable preliminary insights, cellular phenotypes can be confounded by pleiotropic effects, off-target interactions, or non-specific cytotoxicity. Therefore, definitive validation of target-specific mechanisms—such as cellular target-engagement assays (e.g., cellular thermal shift assay), genetic modulation studies (e.g., targeted gene knockdown or knockout), or appropriate mechanistic controls—is fully warranted and constitutes an essential objective for ongoing, subsequent investigations to bridge computational models with rigorous in vitro proof. Building upon these preliminary insights, it is important to recognise that observed variations in cellular efficacy cannot be attributed solely to physicochemical parameters, membrane permeability, or concluded PTP1B target engagement without direct intracellular validation. While in silico ADMET profiling and isolated enzyme assays provide essential predictive guidance, cellular phenotypes are naturally subject to pleiotropic effects, non-specific cytotoxicity, or off-target pathways. To definitively bridge computational predictions with mechanistic in vitro proof, future work should prioritise target-specific validation strategies. Specifically, incorporating cellular target-engagement assays (such as CETSA [53]), targeted genetic modulation (e.g., siRNA knockdown [54] or CRISPR/Cas9 knockout of PTP1B [55]), and appropriate mechanistic controls to establish unambiguous, target-mediated pharmacological responses.

## 4. Conclusions

ADMET profiling is becoming an invaluable, reliable, and highly useful tool that should be employed in the design process of any new drug, not just those intended for cancer treatment. The present study demonstrated that the results of such analyses often signal a need for further molecule optimisation, even when prior in vitro studies using isolated enzyme or cancer cell lines have yielded highly promising results. In silico methods like ADMET profiling do not replace in vitro studies; rather, they complement them by providing a retrospective perspective that contextualises researchers’ findings. They also offer a broader outlook—particularly when an active molecule with potential selectivity for cancer cell lines has been developed—by helping to detect potential toxicity before undertaking synthesis and subsequent costly stages of drug development. This is especially relevant given that the healthy cell lines typically studied often exhibit a limited spectrum of enzymatic activity, making it impossible to accurately assess the potential for toxic metabolite formation at every stage of such metabolic processes. ADMET profiling tools can help overcome this limitation.

However, it is important to be aware of the limitations characteristic of these tools. While they generally show a high degree of concordance between results, the specific datasets used to train them inevitably influence the outcomes. This is particularly evident in toxicity analysis, where the level of concordance is lowest. It is therefore advisable to employ more than one tool to ensure cross-validation of the results. Both ADMETlab 3.0 and admetSAR 3.0 are ideally suited for this purpose. They offer a similarly broad panel of analysed endpoints and proven reliability while relying on different methodologies; the resulting convergence enhances the confidence in the obtained findings. Given that the predictive performance can vary significantly depending on the specific endpoint, while in silico findings justify utilising multiple computational tools for initial screening and hazard identification, they do not inherently guarantee high predictive accuracy across all ADMET parameters. Consequently, consensus among different software tools must be strictly distinguished from experimentally validated accuracy.

In summary, the reliability of ADMET profiling tools can currently be regarded as highly adequate and reliable for predictive screening, whereas the continuous and rapid development of in silico methods suggests they will play an increasingly significant role in the future of drug development.

## Figures and Tables

**Table 1:** List of the analysed compounds with relative IUPAC and source article names.

Compound ID	IUPAC Name (With Name From Source Article in Brackets)	Reference
1	3-Cinnamoyl-5-((E)-4-hydroxy-3-methoxybenzylidene)-1-methylpiperidin-4-one (5)	[39]
2	4-((E)-(5-Cinnamoyl-1-methyl-4-oxopiperidin-3-ylidene)methyl)-2-methoxyphenyl methyl carbonate (6)	[39]
3	2-Acetyl-4-fluoro-6-nitrophenyl 2-fluorobenzenesulfonate (2a)	[40]
4	2-Acetyl-4-fluoro-6-nitrophenyl 4-(trifluoromethoxy)benzenesulfonate (2e)	[40]
5	2-Acetyl-4-chloro-6-nitrophenyl 4-methoxybenzenesulfonate (2f)	[40]
6	2-Acetyl-4-chloro-6-nitrophenyl 2-fluorobenzenesulfonate (2g)	[40]
7	2-Acetyl-4-chloro-6-nitrophenyl 4-(trifluoromethyl)benzenesulfonate (2j)	[40]
8	2-Acetyl-4-chloro-6-nitrophenyl 4-(trifluoromethoxy)benzenesulfonate (2k)	[40]
9	2-Acetyl-4-methyl-6-nitrophenyl 4-(trifluoromethyl)benzenesulfonate (2v)	[40]
10	2-Acetyl-4-methyl-6-nitrophenyl 4-methoxybenzenesulfonate (2x)	[40]
11	5,7,3′,4′-tetramethylquercetin (5)	[41]
12	5,7,4′-trimethylquercetin (6)	[41]
13	5,7,3′,6′-tetramethoxyflavone (7)	[41]
14	(2S)-5,6,7,3′,4′-pentamethoxyflavanone (9)	[41]
15	(2S)-5′-hydroxy-5,7,3′,4′-tetramethoxyflavanone (10)	[41]
16	Methyl 2-hydroxy-5-(1-(2-(4-p-tolylthiazol-2-yl)hydrazono)ethyl)benzoate (3e)	[42]
17	Methyl 5-(1-(2-(4-(4-(fluorosulfonyl)phenyl)thiazol-2-yl)hydrazono)ethyl)-2-hydroxybenzoate (3g)	[42]
18	Methyl 5-(1-(2-(4-(3,4-dihydro-2H-benzo[b][1,4]dioxepin-7-yl)thiazol-2-yl)hydrazono)ethyl)-2-hydroxybenzoate (3j)	[42]
19	Methyl 5-(1-(2-(4-(1H-indol-7-yl)thiazol-2-yl)hydrazono)ethyl)-2-hydroxybenzoate (3l)	[42]
20	5,7-dihydroxy-2-hydroxy-2-[(8Z,11Z,14Z)-hexadeca-8,11,14-trien-1-yl]chroman-4-one (1)	[43,44]
21	5,7-dihydroxy-2-hydroxy-2-[(8Z,11Z,14Z)-hexadeca-8,11,14-trien-1-yl]-6,8-dimethylchroman-4-one (2)	[43,44]
22	5,7-dihydroxy-2-hydroxy-2-[(8Z,11Z,14Z)-hexadeca-8,11,14-trien-1-yl]-8-methylchroman-4-one (3)	[43,44]
23	5,7-dihydroxy-2-hydroxy-2-[(8Z,11Z,14Z)-hexadeca-8,11,14-trien-1-yl]-6-methylchroman-4-one (4)	[43,44]
24	5,7-dihydroxy-2-hydroxy-2-[(9Z,12Z)-hexadeca-9,12-dien-1-yl]chroman-4-one (5)	[43,44]
25	5,7-dihydroxy-2-hydroxy-2-[(9Z,12Z)-hexadeca-9,12-dien-1-yl]-6,8-dimethylchroman-4-one (6)	[43,44]
26	5,7-dihydroxy-2-hydroxy-2-[(9Z,12Z)-hexadeca-9,12-dien-1-yl]-8-methylchroman-4-one (7)	[43,44]
27	5,7-dihydroxy-2-hydroxy-2-[(9Z,12Z)-hexadeca-9,12-dien-1-yl]-6-methylchroman-4-one (8)	[43,44]
28	2-hexadecyl-5,7-dihydroxy-2-hydroxychroman-4-one (9)	[43,44]
29	2-hexadecyl-5,7-dihydroxy-2-hydroxy-6,8-dimethylchroman-4-one (10)	[43,44]
30	2-hexadecyl-5,7-dihydroxy-2-hydroxy-8-methylchroman-4-one (11)	[43,44]
31	2-hexadecyl-5,7-dihydroxy-2-hydroxy-6-methylchroman-4-one (12)	[43,44]
32	5,7-dihydroxy-2-hydroxy-2-[(11Z)-pentadec-11-en-1-yl]chroman-4-one (13)	[43,44]
33	5,7-dihydroxy-2-hydroxy-6,8-dimethyl-2-[(11Z)-pentadec-11-en-1-yl]chroman-4-one (14)	[43,44]
34	5,7-dihydroxy-2-hydroxy-8-methyl-2-[(11Z)-pentadec-11-en-1-yl]chroman-4-one (15)	[43,44]
35	5,7-dihydroxy-2-hydroxy-6-methyl-2-[(11Z)-pentadec-11-en-1-yl]chroman-4-one (16)	[43,44]

**Table 2:** Physicochemical properties of the analysed compounds.

Compound ID	Physicochemical Properties									Isolated PTP1B IC_50_ [µM] (or % of Inhibition)	MCF-7 IC_50_ [µM]	MDA-MB-231 IC_50_ [µM]	Non-cancerous Control Cell Line IC_50_ [µM]
	MW			log*P*			TPSA						
	ADMETlab 3.0	admetSAR 3.0	SwissADME	ADMETlab 3.0	admetSAR 3.0	SwissADME	ADMETlab 3.0	admetSAR 3.0	SwissADME				
1	377.16	377.44	377.43	2.55	3.20	2.93	66.84	66.84	66.84	N/A	25.30 ± 2.54	31.86 ± 1.07	41.24 ± 3.22 (HaCaT)
2	435.17	435.48	435.47	2.47	3.64	3.17	82.14	82.14	82.14	36%	30.51 ± 4.11	11.80 ± 1.43	30.84 ± 7.63 (HaCaT)
3	357.01	357.29	357.29	1.79	2.84	2.72	103.58	103.58	114.64	6.3 ±0.029	4.96 ± 0.05	N/A	39.21 ± 0.13 (Vero)
4	423	423.30	423.29	3.24	3.60	3.38	112.81	112.81	123.87	23.1 ±0.006	8.18 ± 0.03	N/A	20.78 ± 0.14 (Vero)
5	369.03	369.33	369.32	2.17	2.71	2.3	112.81	112.81	123.87	5.9 ±0.018	4.38 ± 0.07	N/A	24.03 ± 1.01 (Vero)
6	372.98	373.75	373.74	2.56	3.36	2.96	103.58	103.58	114.64	16.9 ±0.016	9.92 ± 0.09	N/A	33.93 ± 0.15 (Vero)
7	422.98	423.75	423.75	3.58	4.24	3.79	103.58	103.58	114.64	19.6 ±0.042	2.58 ± 0.02	N/A	38.86 ± 0.16 (Vero)
8	438.97	439.75	439.75	3.91	4.12	3.62	112.81	112.81	123.87	20.1 ±0.065	34.53 ± 0.12	N/A	33.39 ± 0.13 (Vero)
9	403.03	403.33	403.33	3.36	3.89	3.6	103.58	103.58	114.64	7.6 ±0.070	8.58 ± 0.07	N/A	48.02 ± 0.18 (Vero)
10	365.06	365.36	365.36	2.42	2.88	2.28	112.81	112.81	123.87	21.5 ±0.066	11.01 ± 0.07	N/A	56.77 ± 0.18 (Vero)
11	358.11	358.35	358.34	2.21	3.20	2.05	87.36	87.36	87.36	9.76 ± 0.67	28.4 ± 1.6	15.8 ± 0.2	N/A
12	344.09	344.32	344.32	1.95	2.90	1.7	98.36	98.36	98.36	10.12 ± 1.09	22.9 ± 0.6	>30	N/A
13	342.11	342.35	342.34	2.41	3.49	2.37	67.13	67.13	67.13	>30	25.6 ± 1.3	>30	N/A
14	374.14	374.39	374.38	2.72	3.44	2.67	72.45	72.45	72.45	6.88 ± 0.76	11.5 ± 0.7	17.6 ± 1.2	N/A
15	360.12	360.36	360.36	2.71	3.13	2.32	83.45	83.45	83.45	8.92 ± 0.72	15.4 ± 1.6	21.3 ± 0.9	N/A
16	381.11	381.46	381.45	5.15	4.45	4.25	83.81	83.81	112.05	6.91 ± 1.25	24.08 ± 9.19	N/A	10.20 ± 3.52 (HSF)
17	449.05	449.49	449.48	4.61	3.80	3.82	117.95	117.95	154.57	6.86 ± 2.42	26.31 ± 12.74	N/A	40.50 ± 18.71 (HSF)
18	423.13	423.49	423.48	4.35	5.20	3.98	93.04	93.04	121.28	0.51 ± 0.15	21.21 ± 8.52	N/A	231.3 ± 27.44 (HSF)
19	406.11	406.47	406.46	4.69	4.62	3.94	99.6	99.6	127.84	3.18 ± 2.03	20.77 ± 3.27	N/A	203.50 ± 82.71 (HSF)
20	428.26	428.57	428.56	7.09	7.89	5.69	86.99	86.99	86.99	0.45 ± 0.03	29.80 ± 1.70	34.23 ± 0.34	N/A
21	456.29	456.62	456.61	7.65	8.16	6.39	86.99	86.99	86.99	1.6 ± 0.09	56.46 ± 1.60	47.67 ± 0.60	N/A
22	442.27	442.60	442.59	7.43	8.05	5.99	86.99	86.99	86.99	1.3 ± 0.07	30.98 ± 0.67	21.44 ± 0.12	N/A
23	442.27	442.60	442.59	7.36	8.16	5.98	86.99	86.99	86.99	1.3 ± 0.08	30.98 ± 0.67	21.44 ± 0.12	N/A
24	430.27	430.59	430.58	7.50	8.19	5.88	86.99	86.99	86.99	0.42 ± 0.03	23.00 ± 0.22	18.47 ± 0.36	N/A
25	458.3	458.64	458.63	8.05	8.38	6.63	86.99	86.99	86.99	2.7 ± 0.14	53.01 ± 0.26	42.77 ± 0.90	N/A
26	444.29	444.61	444.60	7.85	8.26	6.3	86.99	86.99	86.99	3.2 ± 0.17	49.16 ± 1.45	48.06 ± 0.62	N/A
27	444.29	444.61	444.60	7.79	8.39	6.27	86.99	86.99	86.99	3.2 ± 0.18	49.16 ± 1.45	48.06 ± 0.62	N/A
28	406.27	406.56	406.56	6.59	8.41	5.75	86.99	86.99	86.99	2.0 ± 0.11	20.50 ± 1.37	17.79 ± 0.36	N/A
29	434.3	434.62	434.61	7.46	8.54	6.4	86.99	86.99	86.99	N/A	N/A	N/A	N/A
30	420.29	420.59	420.58	7.00	8.46	6.11	86.99	86.99	86.99	0.98 ± 0.06	18.89 ± 1.03	18.74 ± 0.21	N/A
31	420.29	420.59	420.58	7.00	8.56	6.11	86.99	86.99	86.99	0.98 ± 0.07	18.89 ± 1.03	18.74 ± 0.21	N/A
32	404.26	404.55	404.54	7.00	8.09	5.47	86.99	86.99	86.99	N/A	18.73 ± 2.36	33.93 ± 0.43	N/A
33	432.29	432.60	432.59	7.51	8.14	6.13	86.99	86.99	86.99	1.8 ± 0.10	17.47 ± 1.75	6.79 ± 0.05	46.05 ± 0.01 (HUVEC)
34	418.27	418.57	418.57	7.34	8.22	5.78	86.99	86.99	86.99	1.0 ± 0.06	18.13 ± 0.52	18.96 ± 0.31	N/A
35	418.27	418.57	418.57	7.29	8.23	5.77	86.99	86.99	86.99	1.0 ± 0.07	18.13 ± 0.52	18.96 ± 0.31	N/A

Abbreviations used: MW—molecular weight; log*P*—logarithm of the partition coefficient; TPSA—topological polar surface area. N/A: Experimental data not determined or not reported in the original primary literature. These compounds were retained to maintain the structural diversity of the 35-inhibitor library for in silico ADMET profiling.

**Table 3:** Statistical analysis of correlations between results estimated with evaluated tools for log*P* and TPSA parameters.

Parameter	Predictive Tool	Pearson’s *r*	Coefficient of Determination (*r*^2^)	Significance Test for Pearson’s *r*		Spearman Rank Correlation (*ρ*)
				Student’s *t*	Two-Tailed *p*-Value	
log*P*	ADMETlab 3.0 and admetSAR 3.0	0.976157	0.952883	25.833656	<0.0001	0.877820
	ADMETlab 3.0 and SwissADME	0.983900	0.968059	31.625011	<0.0001	0.957478
	admetSAR 3.0 and SwissADME	0.974332	0.949323	24.863278	<0.0001	0.927086
TPSA	ADMETlab 3.0 and admetSAR 3.0	1.000000	1.000000	∞	<0.0001	1.000000
	ADMETlab 3.0 and SwissADME	0.911407	0.830664	12.723144	<0.0001	0.929503
	admetSAR 3.0 and SwissADME	0.911407	0.830664	12.723144	<0.0001	0.929503

**Table 4:** Statistical analysis of correlations between results estimated with evaluated tools for Caco-2 permeability parameter.

Parameter	Predictive Tool	Pearson’s *r*	Coefficient of Determination (*r*^2^)	Significance Test for Pearson’s *r*		Spearman Rank Correlation (*ρ*)
				Student’s *t*	Two-Tailed *p*-Value	
Caco-2 permeability	ADMETlab 3.0 and admetSAR 3.0	0.623847	0.389185	4.585434	<0.0001	0.514426

**Table 5:** Contingency table for HIA prediction results: SwissADME and ADMETLab 3.0.

	SwissADME	High	Low	Total
ADMETLab 3.0				
High		3	2	5
Low		11	19	30
Total		14	21	35

Number of observed agreements (*p*_o_) = 22 (62.86% of the observations); number of agreements expected by chance (*p*_e_) = 20 (57.14% of the observations); kappa (*κ*) = 0.133; SE of kappa = 0.141; 95% confidence interval: from −0.144 to 0.411; result = slight agreement.

**Table 6:** Contingency table for HIA prediction results: SwissADME and admetSAR 3.0.

	SwissADME	High	Low	Total
admetSAR 3.0				
High		14	21	35
Low		0	0	0
Total		14	21	35

Number of observed agreements (*p*_o_) = 14 (40.00% of the observations); number of agreements expected by chance (*p*_e_) = 14 (40.00% of the observations); kappa (*κ*) = 0.000; result = lack of agreement beyond chance levels, due to the model’s constant prediction.

**Table 7:** Contingency table for HIA prediction results: admetSAR 3.0 and ADMETLab 3.0.

	admetSAR	High	Low	Total
3.0 ADMETLab 3.0				
High		5	0	5
Low		30	0	30
Total		35	0	35

Number of observed agreements (*p*_o_) = 5 (14.29% of the observations); number of agreements expected by chance (*p*_e_) = 5 (14.29% of the observations); kappa (*κ*) = 0.000; result = lack of agreement beyond chance levels, due to the model’s constant prediction.

**Table 8:** Contingency table for Ames test results: admetSAR 3.0 and ADMETLab 3.0.

	admetSAR	High	Low	Total
3.0 ADMETLab3.0				
High		13	4	17
Low		7	11	18
Total		20	15	35

Number of observed agreements (*p*_o_) = 24 (68.57% of the observations); number of agreements expected by chance (*p*_e_) = 17.4 (49.80% of the observations); kappa (*κ*) = 0.374; SE of kappa = 0.154; 95% confidence interval: from 0.072 to 0.676; result = fair agreement.

**Table 9:** Statistical analysis of correlations between results estimated with ADMETlab 3.0 and admetSAR 3.0 for toxicity parameters.

Parameter	Pearson’s *r*	Coefficient of Determination (*r*^2^)	Significance Test for Pearson’s *r*		Spearman Rank Correlation (*ρ*)	Strength (Pearson/ Spearman)
			Student’s *t*	Two-Tailed *p*-Value		
AMES	0.363177	0.131898	2.239187	0.03200	0.452241	moderate/moderate
DILI	0.973687	0.948066	24.544237	0.00010	0.920239	very strong/very strong
hERG 10 µM	0.803299	0.645290	7.748142	0.00010	0.677677	very strong/strong
NR-AR	0.164612	0.027097	0.958700	0.34468	0.042452	weak/very weak
NR-ER	−0.235548	0.055483	−1.392293	0.17315	−0.266816	weak/weak
AMES	0.363177	0.131898	2.239187	0.03200	0.452241	moderate/moderate

## Data Availability

Data is contained within the article and Supplementary Material.

## Supplementary Materials

The following supporting information can be downloaded at https://www.mdpi.com/article/10.3390/biology15212024/s1, Table S1: Number of medicinal chemistry alerts generated by the analysed compounds; Table S2: Comparison of Caco-2 permeability test results and human intestinal absorption (HIA) across ADMET profiling tools; Table S3: Selectivity indexes (SI) of the analysed compounds; Table S4: Results of the toxicity analyses for the analysed compounds.

## Abbreviations

The following abbreviations are used in this manuscript:

ADMET	Absorption, Distribution, Metabolism, Excretion, and Toxicity
AI	Artificial Intelligence
ALARM NMR	A La Assay to Detect Reactive Molecules by Nuclear Magnetic Resonance
Caco-2	Human Epithelial Colorectal Adenocarcinoma Cell Line
CETSA	Cellular Thermal Shift Assay
DILI	Drug-Induced Liver Injury
hERG	Human Ether-à-go-go-Related Gene
HIA	Human Intestinal Absorption
HSF	Human Skin Fibroblasts
HUVECs	Human Umbilical Vein Endothelial Cells
IC_50_	Half-Maximal Inhibitory Concentration
IUPAC	International Union of Pure and Applied Chemistry
log*P*	Logarithm of the Partition Coefficient
MCF-7	Michigan Cancer Foundation-7 Cell Line
MW	Molecular Weight
NR-AR	Nuclear Receptor-Androgen Receptor
NR-ER	Nuclear Receptor-Estrogen Receptor
PAINS	Pan-Assay Interference Compounds
PTP1B	Protein Tyrosine Phosphatase 1B
SI	Selectivity Index
siRNA	Small Interfering Ribonucleic Acid
TPSA	Topological Polar Surface Area
