# Supplementary material for: How Reliable Is In Silico ADMET Profiling? A Comparison of Tools Complementing Experimental In Vitro Analyses of Newly Developed Breast Cancer Therapeutics
Source: Biology (Basel). 2026 Nov 6;15(21):2024. doi: 10.3390/biology15212024 (PMC13762100; doi:10.3390/biology15212024)
Supplement: Supplementary file 1 [file biology-15-02024-s001.zip › biology-4644740-supplementary.pdf]

Table S1. Number of medicinal chemistry alerts generated by the analysed compounds.

Compound ID	PAINS	ALARM NMR
1	0	3
2	0	2
3	0	3
4	0	3
5	0	3
6	0	3
7	0	3
8	0	3
9	0	3
10	0	3
11	0	2
12	0	3
13	0	2
14	0	2
15	0	3
16	1	4
17	1	5
18	1	4
19	1	4
20	0	1
21	0	1
22	0	1
23	0	1
24	0	1
25	0	1
26	0	1
27	0	1
28	0	1
29	0	1
30	0	1
31	0	1
32	0	1
33	0	1
34	0	1
35	0	1

Table S2. Comparison of Caco-2 permeability test results and human intestinal absorption (HIA) across ADMET profiling tools.

Compound ID	Caco-2		HIA		
	ADMETlab 3.0	admetSAR 3.0	ADMETlab 3.0	admetSAR 3.0	SwissADME
1	-5.29	-4.62	0.00	0.98	High
2	-5.04	-5.06	0.00	0.96	High
3	-4.71	-4.66	0.00	0.96	High
4	-4.72	-4.62	0.00	0.96	Low
5	-4.68	-4.61	0.00	0.98	High
6	-4.74	-4.63	0.00	0.97	High
7	-4.68	-4.48	0.00	0.95	Low
8	-4.72	-4.56	0.00	0.96	Low
9	-4.62	-4.48	0.00	0.97	Low
10	-4.87	-4.68	0.00	0.98	High
11	-5.10	-4.34	0.77	0.98	High
12	-5.26	-5.04	0.62	0.96	High
13	-4.49	-4.36	0.21	0.98	High
14	-4.59	-4.07	0.00	0.99	High
15	-4.91	-4.66	0.00	0.99	High
16	-5.35	-4.92	0.00	0.97	High
17	-5.71	-5.03	0.00	0.96	Low
18	-4.99	-5.13	0.00	0.98	Low
19	-5.49	-5.05	0.00	0.97	Low
20	-5.20	-5.17	0.01	0.80	Low
21	-5.05	-5.03	0.00	0.75	Low
22	-5.07	-5.12	0.04	0.78	Low
23	-5.11	-5.04	0.00	0.79	Low
24	-5.18	-4.94	0.00	0.82	Low
25	-5.05	-4.84	0.00	0.75	Low
26	-5.05	-4.93	0.01	0.80	Low
27	-5.08	-4.86	0.00	0.80	Low
28	-5.15	-4.56	0.85	0.84	High
29	-5.06	-4.58	0.51	0.76	Low
30	-5.05	-4.56	0.94	0.81	Low
31	-5.08	-4.53	0.15	0.81	Low
32	-5.15	-4.76	0.05	0.84	High
33	-5.03	-4.72	0.01	0.75	Low
34	-5.04	-4.76	0.13	0.80	Low
35	-5.05	-4.71	0.00	0.81	Low

Table S3. Categorised selectivity indexes (SI) of the analysed compounds.

Compound ID	Categorised SI
1	Low
2	Low
3	Moderate
4	Moderate
5	Moderate
6	Moderate
7	High
8	Low
9	Moderate
10	Moderate
11	N/A
12	N/A
13	N/A
14	N/A
15	N/A
16	Low
17	Low
18	High
19	Moderate
20	N/A
21	N/A
22	N/A
23	N/A
24	N/A
25	N/A
26	N/A
27	N/A
28	N/A
29	N/A
30	N/A
31	N/A
32	N/A
33	Moderate
34	N/A
35	N/A

SI > 10: high selectivity; SI = 2– 10: moderate selectivity; SI < 2: low selectivity

Table S4. Results of the toxicity analyses for the analysed compounds.

Compound ID	AMES		DILI		hERG 10 uM		NR-AR		NR-ER		mitochondrial	
	ADMETlab 3.0	admetSAR 3.0	ADMETlab 3.0	admetSAR 3.0	ADMETlab 3.0	admetSAR 3.0	ADMETlab 3.0	admetSAR 3.0	ADMETlab 3.0	admetSAR 3.0	ADMETlab 3.0	admetSAR 3.0
1	0.56	0.58	0.45	0.69	0.55	0.28	0.00	0.08	0.73	0.16	0.99	0.17
2	0.58	0.25	0.78	0.72	0.52	0.37	0.00	0.21	0.42	0.11	0.97	0.39
3	0.65	0.64	1.00	0.90	0.24	0.11	0.29	0.42	0.55	0.11	0.99	0.89
4	0.20	0.52	1.00	0.87	0.47	0.24	0.03	0.45	0.84	0.22	1.00	0.91
5	0.67	0.57	1.00	0.84	0.33	0.21	0.10	0.24	0.83	0.06	1.00	0.89
6	0.61	0.65	1.00	0.89	0.29	0.12	0.21	0.43	0.55	0.15	0.99	0.92
7	0.25	0.62	1.00	0.86	0.50	0.26	0.08	0.40	0.85	0.24	1.00	0.93
8	0.19	0.55	1.00	0.86	0.55	0.29	0.02	0.46	0.83	0.26	1.00	0.93
9	0.28	0.73	1.00	0.85	0.44	0.32	0.11	0.46	0.88	0.23	1.00	0.92
10	0.69	0.55	1.00	0.80	0.34	0.52	0.11	0.28	0.86	0.07	1.00	0.83
11	0.56	0.92	0.80	0.80	0.47	0.69	0.50	0.89	0.84	0.82	0.89	0.83
12	0.57	0.60	0.79	0.57	0.48	0.34	0.24	0.53	0.76	0.77	0.98	0.94
13	0.71	0.77	0.87	0.73	0.49	0.73	0.16	0.64	0.75	0.22	0.36	0.57
14	0.65	0.70	0.94	0.71	0.50	0.45	0.12	0.41	0.15	0.11	0.35	0.68
15	0.68	0.51	0.92	0.67	0.48	0.34	0.07	0.54	0.15	0.52	0.81	0.82
16	0.79	0.36	0.99	0.74	0.49	0.32	0.00	0.34	0.44	0.53	0.97	0.97
17	0.37	0.35	1.00	0.76	0.47	0.16	0.00	0.17	0.30	0.33	0.83	0.97
18	0.87	0.50	1.00	0.81	0.40	0.40	0.00	0.43	0.40	0.69	0.99	0.97
19	0.88	0.46	1.00	0.70	0.41	0.52	0.00	0.49	0.28	0.74	0.99	0.97
20	0.80	0.08	0.00	0.24	0.74	0.86	0.00	0.36	0.07	0.54	0.97	0.90
21	0.78	0.06	0.00	0.23	0.68	0.79	0.00	0.32	0.01	0.45	0.99	0.90
22	0.80	0.07	0.00	0.22	0.71	0.84	0.00	0.35	0.01	0.49	0.99	0.90
23	0.81	0.07	0.00	0.25	0.70	0.82	0.00	0.34	0.02	0.52	0.99	0.90
24	0.28	0.07	0.00	0.27	0.74	0.82	0.00	0.31	0.59	0.53	1.00	0.92
25	0.26	0.06	0.00	0.23	0.68	0.76	0.00	0.24	0.10	0.39	1.00	0.91
26	0.27	0.06	0.00	0.24	0.71	0.80	0.00	0.28	0.17	0.45	1.00	0.92
27	0.29	0.07	0.00	0.27	0.70	0.78	0.00	0.27	0.29	0.48	1.00	0.91
28	0.14	0.04	0.12	0.29	0.82	0.69	0.61	0.20	0.90	0.45	0.94	0.94
29	0.13	0.04	0.10	0.25	0.77	0.65	0.19	0.16	0.41	0.32	0.98	0.91
30	0.14	0.04	0.10	0.26	0.80	0.68	0.29	0.17	0.55	0.36	0.98	0.93
31	0.15	0.05	0.14	0.28	0.79	0.66	0.43	0.18	0.72	0.39	0.97	0.92
32	0.22	0.06	0.00	0.27	0.78	0.77	0.28	0.27	0.87	0.53	0.98	0.93
33	0.20	0.05	0.00	0.24	0.73	0.71	0.05	0.21	0.33	0.38	0.99	0.92
34	0.22	0.05	0.00	0.24	0.75	0.76	0.09	0.23	0.48	0.43	0.99	0.93
35	0.24	0.05	0.00	0.28	0.74	0.74	0.16	0.24	0.65	0.47	0.99	0.93
